# Reply to: *Ralstonia pickettii* bacteremia in hemodialysis patients: a report of two cases

**DOI:** 10.5935/0103-507X.20160083

**Published:** 2016

**Authors:** Darwin Tejera

**Affiliations:** Asociación Española - Montevideo, Uruguay.

Renal disease patients undergoing chronic hemodialysis are vulnerable to infectious diseases. Both the immunocompromised state of chronic renal disease patients and the presence of endovascular structures and devices such as hemodialysis arteriovenous fistulas and central venous catheters create a favorable environment for infectious complications, mainly bacteremia.^(1)^ This study's main point of interest is that it stresses the high level of clinical suspicion that physicians should have when treating patients with intravascular devices and bacteremia.^(2)^ In this context, despite the few cardiovascular symptoms manifested by gram-negative endocarditis, particularly in cases involving endocarditis caused by infrequent and low-virulence microorganisms, a transesophageal echocardiogram-based search for valvular vegetation is suggested for all patients on hemodialysis with bacteremia.^(2)^ In our setting, there were no new cases; however, infectious agents were isolated in the two described patients, and health control and prevention measures were implemented.

Darwin Tejera

Asociación Española - Montevideo, Uruguay.
